# The potential for self-seeding by the coral *Pocillopora* spp. in Moorea, French Polynesia

**DOI:** 10.7717/peerj.2544

**Published:** 2016-11-09

**Authors:** Georgios Tsounis, Peter J. Edmunds

**Affiliations:** Department of Biology, California State University, Northridge, CA, United States

**Keywords:** Connectivity, Self-seeding, Recovery, Larval production, Closed populations, Recruitment limitation

## Abstract

Coral reefs in Moorea, French Polynesia, suffered catastrophic coral mortality through predation by *Acanthaster planci* from 2006 to 2010, and Cyclone Oli in 2010, yet by 2015 some coral populations were approaching pre-disturbance sizes. Using long-term study plots, we quantified population dynamics of spawning *Pocillopora* spp. along the north shore of Moorea between 2010 and 2014, and considered evidence that population recovery could be supported by self-seeding. Results scaled up from study plots and settlement tiles suggest that the number of *Pocillopora* spp. colonies on the outer reef increased 1,890-fold between 2010 and 2014/2015, and in the back reef, 8-fold between 2010 and 2014/2015. Assuming that spawning *Pocillopora* spp. in Moorea release similar numbers of eggs as con-generics in Hawaii, and fertilization success is similar to other spawning corals, the capacity of *Pocillopora* spp. to produce larvae was estimated. These estimates suggest that *Pocillopora* spp. in Moorea produced a large excess of larvae in 2010 and 2014 relative to the number required to produce the recruits found in the back reef and outer reef in 2010 and 2014, even assuming that ∼99.9% of the larvae do not recruit in Moorea. Less than a third of the recruits in one year would have to survive to produce the juvenile *Pocillopora* spp. found in the back and outer reefs in 2010 and 2014/2015. Our first order approximations reveal the potential for *Pocillopora* spp. on the north shore of Moorea to produce enough larvae to support local recruitment and population recovery following a catastrophic disturbance.

## Introduction

Dramatic reductions in population size of tropical scleractinians (Pandolfi et al., 2003), and poor prospects for their long-term survival (Van Hooidonk, Maynard & Planes, 2013), have focused attention on the conditions under which their populations recover from large declines in size (e.g., Bellwood et al., 2004; Hoegh-Guldberg et al., 2007; Hughes et al., 2010). The size of coral populations is commonly assessed through percentage cover of the benthos in planar view (e.g., Hughes, 1994), but this measure of abundance is only loosely coupled to demographically-meaningful population size (i.e., the number of colonies), and has limited value in projecting changes in population size over time (Babcock, 1991; Hughes & Tanner, 2000). Basic demographic properties (e.g., the abundance of coral colonies) need to be quantified to better predict changes in population sizes of corals, yet these features rarely are recorded.

Among the data required to project changes in coral population size are estimates of reproductive features including fecundity, fertilization success, and the likelihood that larvae will form benthic recruits. Among scleractinians, a diverse array of sexual and asexual modes of reproduction support recruitment (Hall & Hughes, 1996; Baird, Guest & Willis, 2011), but following local extirpation by catastrophic disturbances, population recovery is only possible through recruits derived from pelagic larvae originating from sources external to the affected populations (Bode, Bode & Armsworth, 2006; Jones et al., 2009). Therefore, population recovery following large disturbances—for example, caused by bleaching (Baker, Glynn & Riegl, 2008), and coral predators (Hutchins, 1986; Rotjan & Lewis, 2008)—is influenced by the location of corals capable of supplying larvae, and the likelihood that these larvae will reach the affected reef. Larval connectivity among coral populations therefore has been studied for decades (Sammarco & Andrews, 1988; Jones et al., 2009), and considerable effort has been spent evaluating the potential for self-seeding versus connectivity among reefs as alternative hypotheses describing the means by which population recovery might occur (Selkoe & Toonen, 2011). Early work was influenced by the potential for coral larvae to remain close to the natal reef (Sammarco & Andrews, 1988), but growing awareness of their capacity to disperse favored the alternative view that coral larvae travel far from the natal reef to create open populations (Caley et al., 1996). More recently, understanding of coral recruitment has been refined to emphasize the likelihood that coral populations are more closed than was once thought possible (Cowen & Sponaugle, 2009). New studies on connectivity among coral communities are driving further advances, notably to embrace varying degrees of self-seeding versus connectivity depending on physical environmental conditions and the availability of larvae (Kough & Paris, 2015).

Genetic techniques are effective in quantifying the spatio-temporal scales over which coral populations are connected (Selkoe & Toonen, 2011; Costantini et al., 2011), but they are costly and require analytical sophistication. However, insights into the reliance of coral populations on larvae from more distant locations to sustain growth can be gained by calculating their capacity to produce the larvae necessary to support self-seeding (Mumby, 1999; Jones et al., 2009). Where suitable empirical data are scarce, such calculations must rely on assumptions regarding critical demographic processes such as reproduction and recruitment, and therefore the outcomes serve as first order approximations whose accuracy can only be improved by replacing assumptions with data. Such first order approximations can reveal the extent to which self-seeding is a feasible means to support recruitment, but they cannot establish cause-and-effect relationships with either mode of larval supply. Nonetheless, and despite simplifying assumptions inherent in first order approximations, such efforts can be a valuable means to contextualize more sophisticated efforts focused on the same question, particularly when the implications of uncertainty in key assumptions are evaluated (Johnston, Rickett & Jones, 2014). First order approximations also serve an important role in integrating related results to acquire emergent properties, and they can improve future allocation of limiting scientific resources to address pressing research questions.

Moorea, French Polynesia, lends itself to the use of first order approximations focused on coral population growth, because reefs in this location have been extensively studied (e.g., Berumen & Pratchett, 2006; Lenihan et al., 2011), and recently have experienced dramatic declines in size of coral populations caused by an outbreak of the corallivorous seastar *Acanthaster planci* (Kayal et al., 2012) and Cyclone Oli (February 2010). Population recovery of *Pocillopora* spp. has been nearly complete within 5 y (Bramanti & Edmunds, 2016), and therefore it is germane to ask what source populations could provide the larvae necessary to support this recovery? Currently the location of the *Pocillopora* spp. providing the larvae driving the recovery of outer reefs in Moorea is unclear, although potential sources include colonies on the outer reef that survived the recent disturbances, colonies in the back reef where the disturbances had an attenuated effect, and colonies on adjacent islands such as Tahiti. Previous studies of seawater flow demonstrate that all three sources are feasible in terms of larval transport, because seawater on the outer reef is well mixed, both within this habitat and with the back reef (Hench, Leichter & Monismith, 2008), and seawater flows within 3.9–6.5 days between Tahiti and Moorea (Martinez et al., 2007). Examples from several taxa illustrate that pelagic larvae can be transported from the back reef to the outer reef (Leichter et al., 2013), and from Tahiti to Moorea (Magalon, Adjeroud & Veuille, 2005; Martinez et al., 2007; Yasuda et al., 2015). In the present study, we use data from the Moorea Coral Reef LTER (http://mcr.lternet.edu), together with surveys conducted in January 2015, to inform first order approximations of the potential of larvae from spawning *Pocillopora* spp. in Moorea to support local population recovery. Wherever possible, we use empirical data in our calculations, but rely on reasonable assumptions where suitable data are absent. Below we separate our presentation based on what we have measured versus what we have assumed.

## Materials and Methods

The north shore of Moorea is ∼16 km long with an outer reef area of ∼3.15 km^2^ to a depth of 17 m (i.e., ∼200 m wide), and a back reef with a hard-bottom area of ∼4.58 km^2^, as determined from Google Earth (accessed in March 2015). To evaluate the potential for *Pocillopora* spp. populations along this shore to increase through self-seeding, we compared the growth of populations between 2010 and 2014 with their inferred capacity to produce larvae and support local recruitment. Most empirical data came from two sites on the outer and back reefs (http://mcr.lternet.edu), and analyses of coral cover from 2005–2014 were used to contextualize a contrast of *Pocillopora* spp. populations between surveys conducted in April/May 2010 and mostly in April/May 2014. These sites are representative of the north shore (Edmunds & Leichter, 2016; Edmunds et al., 2016), and exploratory dives and additional phototransects show this assertion broadly to be true (PJ Edmunds, 2010, 2014, 2015, unpublished data). Values from the literature were used to estimate fecundity and larval productions, and are described below under assumptions and calculations. It is not our goal to statistically analyze the temporal trends in community structure reported herein, and for such details readers are referred to other papers (Adam et al., 2011; Bramanti & Edmunds, 2016). Instead we focus on utilizing mean values in support of first order approximation exploring the feasibility of self-seeding for *Pocillopora* spp. in Moorea. Pocilloporids in Moorea are represented by species of *Pocillopora*, a very high proportion of which are spawners (only *P. damicornis* is known to brood larvae in this location, and it is rare on the outer reef). *P. verrucosa*, *P. meandrina*, *P. effusus*, *P. woodjonesi*, *P. eydouxi*, *P. damicornis*, and two unknown haplotypes are found in the region (Bosserelle et al., 2014; Edmunds & Leichter, 2016), and of these, the lineage represented by *P. verrucosa* and *P. meandrina* dominates along the north shore of Moorea (Edmunds & Leichter, 2016; PJ Edmunds, 2010, 2014, 2015, unpublished data). Permits for fieldwork were issued by the Haut-commissariat de la République en Polynésie Française (DRRT) (Protocole d’Accueil 2010–2011, 2011–2012, 2012–2013, 2013–2014 to PJE).

### Empirical data

On the outer reef, community structure was quantified annually in April/May from 2005–2014 using photoquadrats (0.5 × 0.5 m, *n* ∼ 40 site^−1^ y^−1^) placed randomly along a 40-m transect at 10-m and 17-m depth at two sites. In the back reef, community structure was quantified annually (also in April/May) along four, 5 m axes centered on five bommies site^−1^ (*n* ∼ 100 photoquadrats site^−1^ y^−1^). In both habitats, sampling occurred at LTER 1 and 2, with additional sampling at three sites in the back reef that were close to LTER 1 and 2. The LTER sites were established in 2005 to sample the north shore with annual frequency, and three additional sites are sampled every 3–4 y along the outer reef of the north shore to explore the generality of the results obtained from LTER 1 and 2. Outer reef photoquadrats were analyzed for benthic cover using CPCe software (Kohler & Gill, 2006) with 200 points randomly located on each image that were scored to evaluate the cover of all scleractinians and *Pocillopora* spp. For the back reef, the same software was used to measure cover of *Pocillopora* spp. in 2010 and 2014, but cover of all scleractinians between 2005 and 2014 was measured by subdividing the photoquadrats to 25 equal squares and scoring them categorically for the dominant spatial occupant. The sum of squares dominated by scleractinians provided a measure of coral cover (with 4% resolution).

*Pocillopora* spp. recruitment was measured using terracotta tiles (15 × 15 × 1 cm) placed horizontally at both sites on the outer reef (10 m and 17 m depth), and at three sites in the back reef (2–3-m depth, *n* = 15–16 tiles site^−1^). Tiles were replaced every 5–7 months in January/February and August/September, and upon recovery, were bleached, dried, and scored for scleractinian recruits. Recruits were identified to family level based on corallite structures, and here the density of pocilloporids is reported. The number of pocilloporid recruits on each tile was standardized to the area of the lower surface (225 cm^2^) where >90% of recruits were found, and the mean recruitment at each sampling within each year summed to estimate annual recruitment.

To gain further insight into the demographic processes mediating population growth of spawning *Pocillopora* spp., the abundances of juvenile *Pocillopora* spp. (colonies ≤ 4 cm diameter) and the abundance of all *Pocillopora* spp. colonies were measured. On the outer reef, juvenile colonies were quantified at 10-m depth during April/May using quadrats (0.5 × 0.5 m) placed at random positions along the same transects used for the photoquadrat sampling. The time-consuming nature of these surveys prevented repeating them at the deeper site (17 m) where the required bottom time on scuba was restricted by safe diving practices. In the back reef, juvenile *Pocillopora* spp. in 2010 were counted in the photoquadrats recorded at LTER 1 and 2, and ∼5 y later (January 2015) the density of juvenile colonies was quantified using quadrats (0.5 × 0.5 m) positioned randomly at three back reef sites near LTER 1 and 2. Finally, the density of *Pocillopora* spp. colonies (regardless of size) was evaluated in 2010 (back reef and outer reef, 10 m and 17 m), 2014 (outer reef, 10 m and 17 m), and January 2015 (back reef). Analyses of *Pocillopora* spp. populations in the back reef were conducted in January 2015 (rather than using photographs recorded in April 2014), because *in situ* counts provide better resolution of small corals than photographs, and we reasoned this was an acceptable trade-off against the ∼10 month discrepancy in sampling date relative to analyses of the outer reef community (in April/May 2014). For the outer reef in 2010 and 2014, and the back reef in 2010, colony densities (no. 1/4 m^−2^) were evaluated using the photoquadrats described above. For the back reef in January 2015, *Pocillopora* spp. densities were evaluated using quadrats (0.5 × 0.5 m) randomly placed at the same three sites where densities of juvenile *Pocillopora* spp. were measured.

### Assumptions and calculations

Using the empirical data described above, the population size, fecundity, and larval production of *Pocillopora* spp. on the north shore of Moorea was estimated. Several reasonable assumptions were necessary to achieve these objectives. First, the abundance of *Pocillopora* spp. recruits along the shore was estimated from the recruits on settlement tiles, which assumes that the density of recruits on tiles and on natural reef surfaces is similar. While this is unlikely to be true (Harriott & Fisk, 1987; Mundy, 2000; Nozawa, Tokeshi & Nojima, 2008), settlement tiles provide a well-established means to quantify relative changes in abundance of recruits over space and time (Mundy, 2000; Penin et al., 2010). Moreover, the density of coral recruits on settlement tiles is correlated with the density of juvenile corals on reef surfaces (Mundy, 2000; Penin et al., 2010), including in the present study (PJE Edmunds, 2010, 2014, 2015, unpublished data), and therefore settlement tiles provide a tractable means to estimate coral recruitment to natural surfaces. On the outer reef, the mean density of *Pocillopora* spp. recruits on tiles at each site (LTER 1 and 2) and depth was averaged between sites and depths. The densities at each sampling within a year (January/February and August/September) then were summed to estimate annual recruitment (corals per 225 cm^2^). This value was scaled proportionately to the area of hard bottom on the outer reef (∼3.15 km^2^), and calculations were completed for 2010 and 2014. For the back reef, the mean density of *Pocillopora* spp. recruits on tiles at each site (LTER 1 and 2) was averaged between sites, and the densities at each sampling within a year (January/February and August/September) summed to estimate annual recruitment (corals per 225 cm^2^). This value was scaled proportionately to the area of hard bottom in the back reef (∼4.58 km^2^), and calculations were completed for 2010 and 2014.

Second, the abundance of juvenile *Pocillopora* spp. on the north shore was estimated from the number of colonies ≤ 4 cm diameter in quadrats. For the outer reef, the mean number of juvenile colonies (number 1/4 m^−2^) scored *in situ* during April/May at 10 m depth at LTER 1 and 2 was averaged between sites, and scaled proportionately to the area of hard bottom in the outer reef to 17-m depth. This approach assumes that the density of juvenile colonies is similar among depths, which is reasonable given the similarity of *Pocillopora* spp. recruitment at 10-m and 17-m depth (e.g., 0.87 corals 225 cm^−2^ versus 0.90 corals 225 cm^−2^, respectively). The abundance of juvenile corals was calculated as described above for 2010 and 2014. For the back reef, in 2010 the mean number of juvenile colonies at LTER 1 and 2 was determined in April/May using photoquadrats, and the mean values by site were averaged (number 1/4 m^−2^), and scaled proportionately to the area of the hard bottom in the back reef. To estimate densities of juvenile corals in the back reef in April/May 2014 (when densities of juvenile corals were estimated on the outer reef), *in situ* surveys were conducted during the next field expedition in January 2015. In situ surveys conducted ∼10 months after the outer reef sampling were used for this purpose, as they provide better resolution of small corals than photographs, and because anecdotal field observations suggested the abundance of juvenile corals broadly were similar in April/May 2014 and in January 2015. Densities of juvenile colonies were averaged by site (number 1/4 m^−2^) and scaled proportionately to the area of the hard bottom in the back reef.

Third, the population size of *Pocillopora* spp. (regardless of size) was estimated as the number of colonies along the north shore. For the outer reef, the mean number of colonies (number 1/4  m^−2^) at LTER 1 and 2 was averaged between sites and depths, and scaled proportionately to the area of hard bottom in the outer reef to 17-m depth. This calculation was completed separately for 2010 and 2014. For the back reef, the mean number of colonies at each site was averaged within each year (number 1/4 m^−2^), and scaled proportionately to the area of the hard bottom in the back reef.

Finally, estimates of local larval production by *Pocillopora* spp. were difficult to generate, as none of the critical pieces of information necessary for this purpose were available for spawning members of this genus in Moorea. The estimation process focused on spawning *Pocillopora* spp., which dominate in Moorea, and was accomplished by estimating the number of sexually mature colonies, fecundity (eggs polyp^−1^), and fertilization success. The size of sexual maturity of *Pocillopora* spp. in Moorea is known only for the uncommon brooder *P. damicornis* (Combosch & Vollmer 2013) and, therefore, this size for spawning taxa was taken as 14-cm diameter, which is the size of sexual maturity in Hawaii for the congeneric spawner *P. meandrina* (Stimson, 1978). The mean size of colonies ≥14-cm diameter was calculated in 2010 and 2014 for the outer reef and back reef, using photoquadrats recorded in April/May at LTER 1 and 2. On the outer reef, logistical constraints limited this analysis to photoquadrats at 10-m depth, and in 2014, the analysis was based on sizes of *P. verrucosa,* which were exceptionally abundant. Given the more rapid growth of corals in shallow compared to deep water, the use of photoquadrats at 10-m depth to estimate the number of sexually mature colonies probably upwardly biased estimates of the number of sexually mature colonies in seawater > 10-m depth, and downwardly biased estimates in seawater < 10-m depth. To estimate the amount of live tissue on the mean-sized, sexually mature *Pocillopora* spp., we used a calibration line (*Y* = 2.493 × *X*^2.312^, *r*^2^ = 0.986) between tissue area (*Y*, cm^2^) and colony diameter (*X*, cm) for *P. verrucosa* collected from 10 m depth in Moorea in April 2014. This calibration was prepared for a separate study, and used wax-dipping (Stimson & Kinzie, 1991) to estimate tissue area (PJ Edmunds, 2010, unpublished data).

Fecundity (egg polyp^−1^) of spawning *Pocillopora* spp. was estimated from three studies reporting 7,300 eggs cm^−2^ y^−1^ (*P. verrucosa* from the Republic of Maldives (Sier & Olive, 1994)), 63 eggs polyp^−1^ (for *P. verrucosa* from the Red Sea (Fadlallah, 1985)) and 114 eggs polyp^−1^ (*P. meandrina* in Hawaii (Stimson, 1976)). The mean fecundity from these studies (6,327 ± 1,882 eggs cm^−2^ (± SD)) was converted to area-normalized fecundity assuming *Pocillopora* spp. have 66 polyps cm^−2^ (Tricas, 1989), and then multiplied by the summed area of the colonies on the north shore that were sexually mature (i.e., ≥ 14-cm diameter).

Nothing is known about the fertilization success of spawning *Pocillopora* spp. in Moorea, therefore we assumed that eggs were fertilized at a rate similar to that reported in two studies of spawning corals—Oliver & Babcock (1992) (*n* = 3 in Fig. 6 for *Montipora digitata*) and Levitan et al. (2004) (*n* = 12 in Fig. 9 for *Orbicella* spp.)—which together indicate a mean fertilization success of 15 ± 21% (±SD).

To evaluate the effects of variability in the estimates of fecundity and fertilization success on population parameters, Monte Carlo sampling (*n* = 20,000) was applied to the calculations, assuming fecundity and fertilization were normally distributed with SD values as reported above. Monte Carlo sampling was conducted using Caladis software (http://www.caladis.org) (Johnston, Rickett & Jones, 2014). The values generated by Monte Carlo sampling were tested for kurtosis and skewness using the moments package (Komsta, 2015) in R software (R Core Team, 2013), and normality was tested using a Kolmogorov–Smirnov test in R software.

## Results

**Figure 1:**
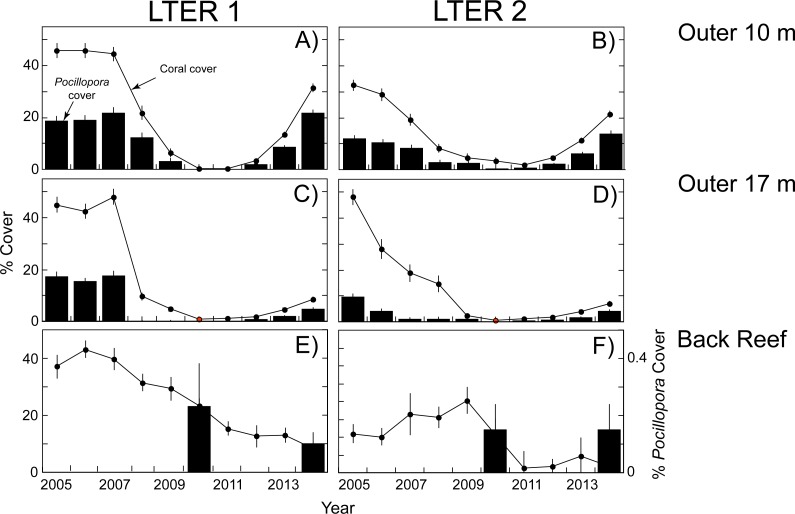
Coral community structure at two sites (LTER 1 and 2) on the north shore of Moorea from 2005–2014. Dots and lines show cover of all scleractinian corals, and bar graphs show cover of *Pocillopora* spp. All values means ± SE (*n* = 37–40). (A, B) Outer reef at 10-m depth, (C, D) Outer reef at 17-m depth, and (E, F) Back reef at 2–5 m depth. For back reef, cover of *Pocillopora* is only available for 2010 and 2014, and the values are scaled against the right ordinate because they are <0.3%.

**Figure 2:**
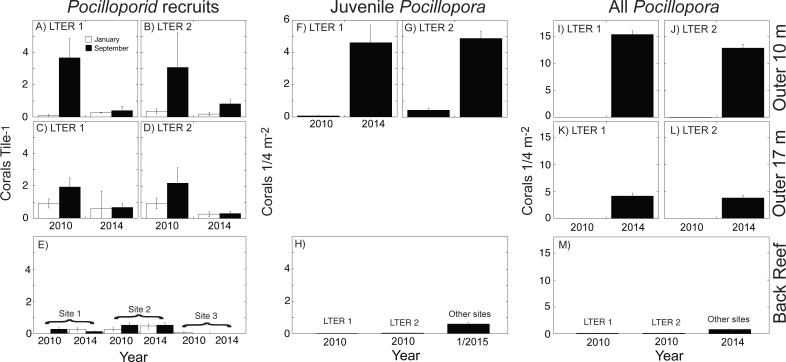
Abundance of three life stages of *Pocillopora* spp. on the north shore of Moorea in 2010, 2014, and January 2015 for pocilloporid recruits (A–E), juvenile *Pocillopora* spp. colonies (F–H), and all *Pocillopora* spp. colonies (I–M). Values shown are mean ± SE for the outer reef at 10-m depth (upper row), outer reef 17-m depth (middle row), and back reef (lower row); data are not available for juvenile corals at 17-m depth. Recruits were censused in January and September of each year, and all other surveys were conducted in April and May. (A–D) recruits at LTER 1 and 2, (E) recruits at three back reef sites in the middle of the north shore, (F, G) juveniles at LTER 1 and 2, (H) juveniles at LTER 1 and 2 in 2010, and at multiple sites in January 2015, (I–L) all *Pocillopora* spp. colonies at LTER 1 and 2, and (M) all *Pocillopora* spp. colonies at LTER 1 and 2 in 2010, and at multiple sites in January 2015.

**Figure 3:**
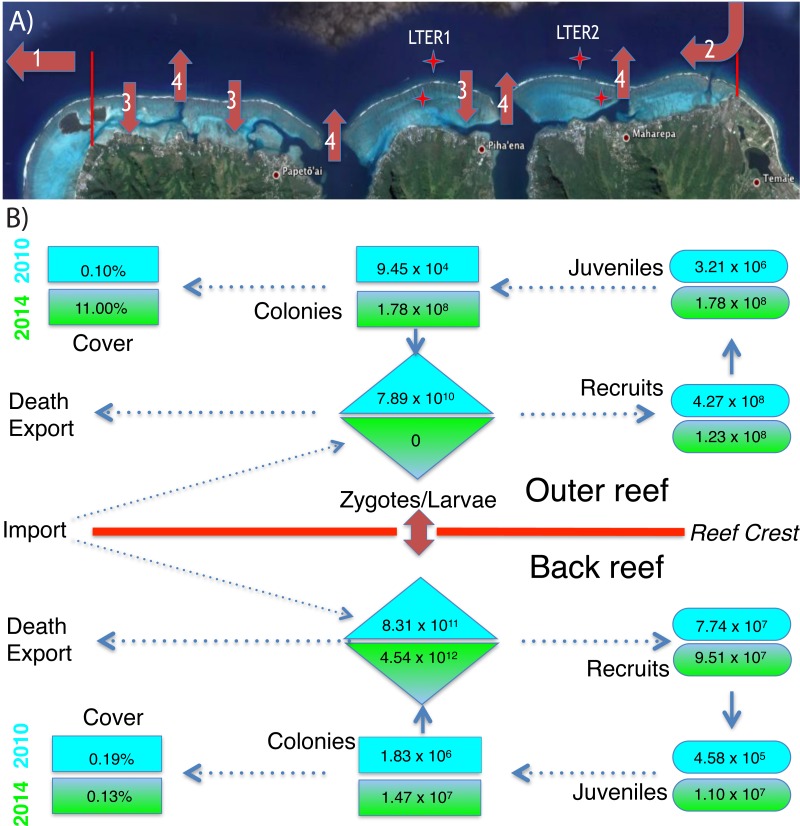
Environmental context and abundance of life stages of *Pocillopora* spp. on the north shore of Moorea. (A) The north shore of Moorea, is ∼16 km long between the red markers, and the back reef extends from the shore to reef crest (∼750 m) with ∼42% hard bottom (Map data ©2015 Google). The outer reef extends seaward of the reef crest, and ∼200 m from the crest the seawater is ∼17 m deep. Coral larvae can die or be exported from the north shore (arrow 1), imported from nearby islands (arrow 2), and travel repeatedly between the outer and back reef habitats (arrows 3 and 4). Stars mark the location of LTER 1 and 2 on outer reef and back reef. (B) The abundance of *Pocillopora* spp. in four demographic stages (zygotes/larvae, recruits, juveniles, and all colonies) and their linkage to percentage cover of this taxon in 2010 (blue) and 2014 (green) for the back reef and outer reef. Abundances of each life stage are calculated as described in the text using values shown in Figs. 1 and 2.

The empirical data used in the present calculations are shown in Figs. 1 and 2. When this study began in 2005, the outer reef of Moorea at 10-m depth had a mean coral cover of 39%, of which 30% was *Pocillopora* spp. (15% cover). At 17-m depth, coral cover was higher (47% cover), and 28% was *Pocillopora* spp. (13% cover). Five years later, predation by *Acanthaster planci* and damage by Cyclone Oli had reduced mean coral cover to 2% at 10-m depth, and 1% at 17-m depth, and *Pocillopora* spp. virtually was absent (cover < 0.2% at both depths). The recovery of coral community structure was well underway 4 y later (2014), when mean coral cover had risen to 27% at 10-m depth, and 8% at 17-m depth, the majority of which (>53%) was *Pocillopora* spp. at 10-m depth (18% cover) and 17-m depth (4% cover). A different pattern was observed in the back reef, where mean coral cover declined from 20% in 2010 to 10% in 2014, and *Pocillopora* spp. was a minor component of this cover. In 2010, mean *Pocillopora* spp. cover was only 0.2% in 2010 and 0.1% in 2014 (Fig. 3).

Following Cyclone Oli in February 2010, recruitment of pocilloporids to tiles on the outer reef was evaluated in September 2010, when overall recruitment (averaged between sites) was 3.37 recruits 225 cm^−2^ at 10-m depth, and 2.06 recruits 225 cm^−2^ at 17-m depth (Figs. 2A–2E). Pocilloporid recruitment was an order of magnitude lower in January 2010, with densities of 0.21 recruits 225 cm^−2^ at 10-m depth, and 0.93 recruits 225 cm^−2^ at 17-m depth (averaged between sites). Together, summed pocilloporid densities in January and September 2010 were 3.58 recruits 225 cm^−2^ at 10-m depth, 2.99 recruits 225 cm^−2^ at 17-m depth, and 3.29 recruits 225 cm^−2^ averaged across depths. Assuming these densities apply to the outer reef along the north shore, recruitment in 2010 brought 4.27 × 10^8^ recruits to the outer reef. Likewise, pocilloporid recruitment at the three back reef sites in 2010 was 0.27 recruits 225 cm^−2^, 0.80 recruits 225 cm^−2^ and 0.07 recruits 225 cm^−2^, so that overall recruitment (averaged among sites) was 0.38 recruits 225 cm^−2^. Assuming this density applies to all hard surfaces, pocilloporid recruitment in 2010 brought 7.74 × 10^7^ recruits to the back reef. Pocilloporid recruitment in the back reef and outer reef in 2010, therefore, brought 5.05 ×10^8^ recruits to the whole reef (i.e., back reef and outer reef). Four years later (2014), densities of recruits on tiles on the outer reef were depressed 71% compared to 2010, with 0.88 recruits 225 cm^−2^ (summed between January and September, and averaged between sites and depths), but densities on tiles in the back reef were elevated 23% compared to 2010, with 0.47 recruits 225 cm^−2^ (summed between January and September, and averaged among sites). Scaling these densities for pocilloporids to the north shore as described above for 2010, indicates that 2014 brought 9.51 ×10^7^ recruits to the back reef, 1.23 × 10^8^ recruits to the outer reef (Fig. 3), and 2.18 × 10^8^ recruits to the whole reef (i.e., back reef and outer reef).

When juvenile *Pocillopora* spp. at 10-m depth on the outer reef were censused in April 2010, densities were low (Figs. 2F–2H). Mean densities at LTER 1 were 0.08 colonies 0.25 m^−2^, and at LTER 2 they were 0.30 colonies 0.25 m^−2^, which together give a mean density of 0.26 colonies 0.25 m^−2^. Assuming this density is representative of the outer reef to 17-m depth, in 2010 there were 3.21 ×10^6^ colonies 0.25 m^−2^ of juvenile *Pocillopora* spp. in this habitat. In the back reef at the same time, mean densities of juvenile *Pocillopora* spp. were 0.02 colonies 0.25 m^−2^ and 0.03 colonies 0.25 m^−2^at LTER 1 and 2, which together give a mean density of 0.02 colonies 0.25 m^−2^. Assuming this density is representative of the hard bottom in the back reef, in 2010 there were 4.58 × 10^5^ colonies 0.25 m^−2^ of juvenile *Pocillopora* spp. in this habitat. Four years later (2014/2015) densities of juvenile *Pocillopora* spp. were higher, with a 19-fold increase in the outer reef to 4.75 colonies 0.25 m^−2^ (averaged between sites), and by January 2015, there was a 24-fold increase in the back reef to 0.60 colonies 0.25 m^−2^ (averaged among sites). These values suggest that in April 2014 there were 5.98 ×10^7^juvenile *Pocillopora* spp. on the outer reef, and in January 2015 there were 1.10 ×10^7^juvenile *Pocillopora* spp. in the back reef (Fig. 3). Overall, therefore, based on surveys conducted in April 2014 and January 2015, there were 2.18 ×10^8^ juvenile *Pocillopora* spp. on the north shore, which is 57% less than 2010.

**Figure 4:**
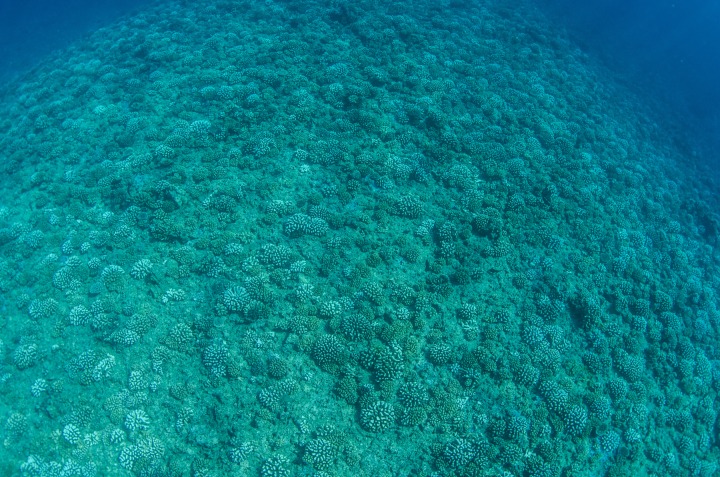
The outer reef on the north shore of Moorea in April 2015. This reef is at ∼12 m depth and the image shows the high density of pocilloporid corals that have grown since 2010.

The size of the *Pocillopora* spp. population (i.e., the number of colonies, regardless of diameter) greatly varied between 2010 and 2014 (Figs. 2I–2M). Surveys conducted in 2010 showed that the mean density of *Pocillopora* spp. colonies was 0.01 colonies 0.25 m^−2^ in the outer reef (averaged between sites and depth), and 0.10 colonies 0.25 m^−2^ in the back reef (averaged between sites), but 4 y later increases in these mean values were large. By 2014 (and relative to 2010), the mean density of *Pocillopora* spp. on the outer reef had increased 1,210-fold to 9.08 colonies 0.25 m^−2^, and by January 2015, in the back reef it had increased 8-fold to 0.80 colonies 0.25 m^−2^. These densities suggest that in 2010, there were 9.45 × 10^4^ *Pocillopora* spp. colonies on the outer reef of the north shore, with an additional 1.83 × 10^6^ in the back reef. These numbers rose to 1.78 × 10^8^ on the outer reef by April 2014, and to 1.47 × 10^7^ in the back reef by January 2015 (Figs. 3 and 4).

The increases in size of *Pocillopora* spp. populations were driven by recruitment of larvae (i.e., new recruits were found). Assuming sexual maturity of spawning *Pocillopora* spp. is obtained at ≥14-cm diameter, in 2010, 21% (*n* = 19 colonies in the quadrats censused) of *Pocillopora* spp. on the outer reef was sexually mature, as were 50% of the colonies in the back reef (*n* = 2 colonies in the quadrats censused). By 2014, these numbers had changed to 0% on the outer reef (*n* = 2,245 colonies of *P. verrucosa* in the quadrats censused), and 10% in the back reef (n = 191 colonies in the quadrats censused). The mean diameter of sexually mature colonies in the back reef was 19.1 cm (*n* = 4) in 2010, and 22.3 cm (*n* = 19) in 2014, and in the outer reef it was 17.1 cm (*n* = 1) in 2010. Using an empirical relationship between diameter and tissue area for *P. verrucosa* in Moorea, and a mean fecundity of 6,327 eggs polyp^−1^ y^−1^, these sexually mature colonies would each release 1.44 ×10^7^eggs cm^−2^, 2.07 × 10^7^ eggs y^−1^, and 1.12 ×10^7^ eggs y^−1^, respectively. Based on the proportion of the *Pocillopora* spp. in the population that was sexually mature (i.e., as estimated from their size), *Pocillopora* spp. on the outer reef of the north shore probably released 5.29 × 10^11^ eggs in 2010, and no eggs in 2014, and in the back reef, they probably released 5.23 × 10^11^ eggs in 2010, and 3.03 × 10^13^ eggs in 2014. If 15% of these eggs are fertilized in the seawater, on the outer reef they would produce 7.89 × 10^10^ zygotes in 2010, and no zygotes in 2014, and in the back reef, 8.30 × 10^11^ zygotes in 2010, and 4.54 × 10^11^ zygotes in 2010. Without information on the proportion of zygotes that develop into competent larvae, the number of zygotes was taken as the best estimate of larval production for the study populations (Fig. 3).

To gain insight into the effects of variation in fecundity and fertilization success on the estimates of larval production, calculations were repeated using Monte Carlo simulations with a range of normally distributed estimates for fecundity and fertilization based on the SDs of the replicate values. These simulations reveal mean (± SD) larval productions in the outer reef of ∼7.89 ×10^10^ ± 1.96 × 10^10^ in 2010, and zero in 2014 (because there was no change in the proportion of colonies that were sexually mature), and in the back reef, 8.31 × 10^11^ ± 2.06 × 10^11^ larvae in 2010, and 4.54 × 10^12^ ± 1.13 × 10^12^ in 2014. The frequency distributions of estimated larval production (*n* = 20,000 for each simulation) were positively skewed (skewness: 0.59 for the back reef in 2010; and in 2014, 0.64 for the outer reef, and 0.64 for the back reef), but leptokurtic (kurtosis: 3.5 for the back reef in 2010; and in 2014, 3.7 for the outer reef, and 3.8 for the back reef; see Komsta, 2015). Therefore, estimates of larval production were tightly distributed around the mean when a range of possible values was considered for fecundity and fertilization success, which are estimated with the greatest uncertainty relative to the values that might be expected for Moorea.

## Discussion

Our calculations estimate the capacity of *Pocillopora* spp. in Moorea to support local recruitment and population growth, but they do not replace genetic tools (e.g., Selkoe & Toonen, 2011) for quantifying connectivity. Instead, our results describe first order approximations using empirical data and reasonable assumptions, and together, they are consistent with the hypothesis that populations of *Pocillopora* spp. on the north shore recovered following recent disturbances through recruitment of larvae produced in this location (i.e., self-seeding). Moreover, our calculations suggest that *Pocillopora* spp. on the north shore had the capacity to export larvae from this shore, even while the outer reef populations were devastated, or in an early recovery stage, and before most colonies of mass spawning members of this genus were sexually mature. Implicit in this conclusion is that *Pocillopora* spp. in the back reef, as well as any large *Pocillopora* spp. colonies that survived the disturbances on the outer reef, produced the larvae necessary to support the ongoing recovery of *Pocillopora* spp. on the outer reef (Bramanti & Edmunds, 2016). Although larvae from colonies in the back reef must reach the outer reef in order to contribute to population growth in this location, this possibility is easily accomplished through the wave-driven, cross-reef transport that characterizes flow regimes in this location (Hench, Leichter & Monismith, 2008).

Our approach has limitations consistent with a first order approximation, but nonetheless, it represents a reasonable summation of available data to realize emergent properties. One important focus of these limitations is uncertainty arising from the complexity of poorly-resolved species within the genus *Pocillopora* (Pinzón et al. 2013; Schmidt-Roach et al., 2014; Edmunds & Leichter, 2016), and the possibility that they exhibit a diversity of reproductive modes (Schmidt-Roach et al., 2012; Schmidt-Roach et al., 2014) with implications for larval production that are more complex than those arising from the assumptions utilized in the present study. In the case of Moorea, however, the implication of these effects may be reduced by the high proportional representation of *P. verrucosa* among *Pocillopora* spp. on the outer reef (Edmunds & Leichter, 2016). Regardless of the merits of first order approaches in biological research (Johnston, Rickett & Jones, 2014), a key limitation of the present calculations is that they cannot show that locally-produced *Pocillopora* spp. larvae recruit locally, or that distantly-produced *Pocillopora* spp. larvae support local recruitment. Both possibilities are supported by different genetic studies of *Pocillopora* spp. in the Society Islands, with one study using samples collected between 2001 and 2003 highlighting connectivity between populations of *P. meandrina* on Moorea and Tahiti (Magalon, Adjeroud & Veuille, 2005), and another study using samples collected in 2013 suggesting that *Pocillopora* spp. in Moorea and Tetiaroa represent unique samplings of genetically discrete larval assemblages (Edmunds & Leichter, 2016). The results of these studies, as well as the present analysis, are not necessarily inconsistent however, because the two studies focus on different aspects of connectivity (adults vs larvae), and connectivity among populations can vary depending on timing of larval release, larval behavior, and hydrodynamic regime at the time of larval availability (Kough & Paris, 2015). Clearly, further application of genetic tools is required to resolve the implications of the present analysis as well as related studies (Magalon, Adjeroud & Veuille, 2005; Edmunds & Leichter, 2016), but regardless of the outcome of these analyses, it is noteworthy that our results are consistent with at least one recent study (Concepcion, Baums & Toonen, 2014). In this analysis, Concepcion, Baums & Toonen (2014) applied genetic tools to study recruitment of mass spawning *Montipora capitata* in the Hawaiian archipelago, and found >90% self-recruitment, even though pelagic larval duration of this species can exceed 200 d.

Of the demographic properties described for *Pocillopora* spp. in Moorea, our estimates of fecundity and fertilization success have the greatest uncertainty, because our calculations rely on values from studies conducted in different locations and at various times. Monte-Carlo simulations using variation about these values provide an indication of the implications of this uncertainty. As expected, these simulations reveal variation in the estimates of larval production by *Pocillopora* spp. in Moorea, but the leptokurtic distribution of these values suggest that variation in fecundity and fertilization success do not greatly affect the present conclusions regarding the capacity of *Pocillopora* spp. to self-recruit.

The history of investigating connectivity among coral populations has changed from favoring self-seeding and short dispersal (Sammarco & Andrews, 1988), to wide dispersal and open populations (Caley et al., 1996), and back to limited dispersal and self-recruitment (Cowen & Sponaugle, 2009; Concepcion, Baums & Toonen, 2014). This reflects the difficulty of quantifying connectivity among separate populations, and clearly there is a great need to use the appropriate tools to quantify self-recruitment for multiple species of corals in a variety of locations and at different times. Regardless of the outcome of analyses that are in the domain of genetic research, the present study takes a different approach using empirical ecological data to shed light on the proximal events that drive variation in population connectivity through larval production. Our first order approximations reveal how large variations in larval supply can arise, for example, when low fecundity and poor fertilization yields minimal numbers of larvae, and when high values for these features generate vast numbers of larvae that potentially saturate local habitats and drive population recovery following catastrophic disturbances. In support of this possibility, analyses of *Pocillopora* spp. recruitment on the north shore of Moorea following recent catastrophic disturbances suggests that *Pocillopora* spp. larvae saturate the outer reef and drive negative density-association recruitment (Bramanti & Edmunds, 2016). This empirical trend underscores our conclusion that the available ecological data cannot be used to reject the hypothesis that even heavily damaged populations of *Pocillopora* spp. may be capable of producing enough larvae to support self-seeding, at least when produced in conjunction with conditions favoring retention, settlement, and post-settlement success. Future studies now are required to test this hypothesis in an inferential framework.

## Additional Information and Declarations

### Data Availability

The following information was supplied regarding data availability: Raw data can be found at the following link: DOI 10.6073/pasta/99465c6f1c1a5d538fafc67f603f00dc.
